# Patient-Reported Quality of Life Predicts First-Cycle Pharmacotherapy Survival in Breast Cancer: A Prospective Cohort Study

**DOI:** 10.3390/medsci14020247

**Published:** 2026-05-10

**Authors:** Henry Sutanto, Merlyna Savitri, Een Hendarsih, Ami Ashariati

**Affiliations:** 1 Internal Medicine Specialist Study Program, Department of Internal Medicine, Faculty of Medicine, Universitas Airlangga, Surabaya 60132, Indonesia; 2 Department of Internal Medicine, Dr. Soetomo General Academic Hospital, Surabaya 60286, Indonesia; merlyna.savitri@gmail.com; 3 Division of Hematology and Medical Oncology, Department of Internal Medicine, Faculty of Medicine, Universitas Airlangga, Surabaya 60132, Indonesia; 4 Division of Hematology and Medical Oncology, Department of Internal Medicine, Haji General Hospital, Surabaya 60116, Indonesia; hendarsih_een@hotmail.com; 5 Integrated Oncology Unit, Universitas Airlangga Hospital, Surabaya 60115, Indonesia

**Keywords:** breast cancer, quality of life, patient reported outcome measures, antineoplastic agents, oncology

## Abstract

Objective: Patient-reported outcomes (PROs) provide direct insight into symptom burden and functional status, yet their utility in early pharmacotherapy outcomes in breast cancer remains underexplored. This study investigated whether baseline PROs from the EORTC QLQ-C30 and BR23 questionnaires are associated with and demonstrate discriminative ability for short-term survival during the first pharmacotherapy cycle in breast cancer patients. Methods: We conducted a prospective cohort study at two secondary referral hospitals in Indonesia from January to October 2025. Women with breast cancer initiating systemic pharmacotherapy were enrolled and followed through completion of the first treatment cycle. The primary outcome was survival of the first cycle, defined as a combined endpoint of death or treatment discontinuation due to adverse effects. Baseline demographic, clinical, and tumor characteristics, along with PROs (EORTC QLQ-C30 and BR23), were compared between survivors (n = 99) and non-survivors (n = 7). Statistical comparisons used Mann–Whitney U tests for PROs. Receiver operating characteristic (ROC) curve analyses were performed to evaluate the discriminative performance of PRO domains. Results: Non-survivors were more likely to present with metastatic disease (71.4% vs. 25.3%). Baseline PROs showed marked differences, with survivors reporting higher global health (83.3 vs. 41.7; *p* < 0.001) and better physical, role, emotional, and social functioning (all *p* < 0.05). Symptom burden—including pain, appetite loss, constipation, systemic side effects, arm symptoms, and financial difficulty—was higher among non-survivors (all *p* < 0.05). ROC analysis demonstrated strong discriminative performance for global health status (AUC 0.907; *p* < 0.001), emotional functioning (AUC 0.846; *p* = 0.002), and social functioning (AUC 0.843; *p* = 0.003), with moderate performance for physical functioning (AUC 0.737; *p* = 0.037). Symptom domains showed lower and inverse AUC values, reflecting scale directionality and limited discriminative capacity. Conclusions: Baseline PROs using EORTC QLQ-C30 and BR23 were strongly associated with early pharmacotherapy survival and demonstrated meaningful discriminative ability, particularly in global and functional domains. Integrating PRO assessments before treatment initiation may enhance early risk stratification, guide supportive care, and improve treatment continuity in breast cancer.

## 1. Introduction

Breast cancer remains the most commonly diagnosed malignancy among women worldwide and a leading cause of cancer-related mortality, with disproportionately greater burdens in low- and middle-income countries (LMICs) [1]. Despite advances in systemic therapy, early discontinuation of pharmacotherapy—driven by toxicity, poor tolerance, and functional decline—continues to impede optimal treatment delivery and compromises survival outcomes [2,3,4]. Identifying patients at risk of early treatment failure remains a considerable challenge in routine oncology practice, particularly in resource-limited settings where supportive care, toxicity monitoring, and multidisciplinary coordination are constrained. Patient-reported outcomes (PROs) have emerged as essential tools for capturing subjective experiences that clinical assessments often fail to detect [5]. Instruments such as the European Organisation for Research and Treatment of Cancer Quality-of-Life Questionnaire-Core 30 (EORTC QLQ-C30) and its breast cancer–specific module (QLQ-BR23) provide validated, multidimensional measures of functioning, symptoms, and global health status directly from the patient’s perspective [6,7]. Accumulating evidence suggests that baseline PROs predict long-term survival, treatment adherence, and toxicity across multiple cancer types [8,9,10]. However, most existing studies have focused on overall survival or progression-related endpoints, with limited attention to the early phase of systemic therapy, where the highest risk of toxicity-related drop-out and early mortality typically occurs [10].

Critically, little is known about whether PROs collected before pharmacotherapy can predict short-term survival of the first treatment cycle, an outcome of major clinical relevance that reflects both biological disease aggressiveness and patients’ functional reserve to withstand systemic therapy. This gap is even more pronounced in LMIC settings and secondary referral hospitals, where resource limitations may amplify the consequences of poor baseline health status and hinder early toxicity management [11]. Evaluating PROs as prognostic markers in such contexts is essential to guide anticipatory supportive care, tailor treatment intensity, and improve early retention in therapy. Given these unmet needs, we conducted a prospective cohort study in two secondary referral hospitals in Indonesia to evaluate whether baseline PROs measured by the EORTC QLQ-C30 and QLQ-BR23 are associated with, and demonstrate discriminative ability for, survival during the first pharmacotherapy cycle, defined as a combined endpoint of death or treatment discontinuation due to adverse effects. By focusing on the earliest phase of treatment, this study provides novel evidence on the potential of PROs to function as rapid prognostic indicators that capture patients’ functional reserve and symptom burden prior to therapy initiation. These findings may inform early risk stratification, guide anticipatory supportive care, and optimize resource allocation in real-world clinical settings. Ultimately, this work supports the integration of PROs into routine clinical practice and underscores their role in improving early pharmacotherapy outcomes in breast cancer.

## 2. Materials and Methods

### 2.1. Study Design and Setting

This prospective cohort study was conducted in two secondary referral hospitals in Indonesia between January and October 2025 [7,12,13]. Both centers serve as major referral facilities for oncology in East Java, managing diverse breast cancer populations from urban and peri-urban catchment areas. The study was designed to evaluate whether baseline PROs are associated with survival across the first cycle of systemic pharmacotherapy. Ethical approval was obtained from the Ethics Committee of both centers (197/KEP/2024 and 445/02/KOM.ETIK/2025). All procedures adhered to the Declaration of Helsinki, and written informed consent was obtained from all participants prior to enrolment.

### 2.2. Sample Size Calculation

The sample size for this study was derived from a larger prospective umbrella cohort investigating PROs in breast cancer pharmacotherapy [7,13]. The initial sample size was calculated using G*Power software version 3.1.9.7 (Heinrich-Heine-Universität Düsseldorf, Düsseldorf, Germany; [14]) based on a paired design framework from the parent study, with an effect size of 0.3, alpha = 0.05, and statistical power (1–β) = 0.8. This yielded a minimum required sample size of 90 participants. The present analysis represents a predefined sub-analysis of early pharmacotherapy outcomes within this cohort. Therefore, no additional sample size calculation was performed specifically for the survival comparison. All eligible participants recruited during the study period were included to maximize available events and statistical power for exploratory analyses.

### 2.3. Participants

**Figure 1:**
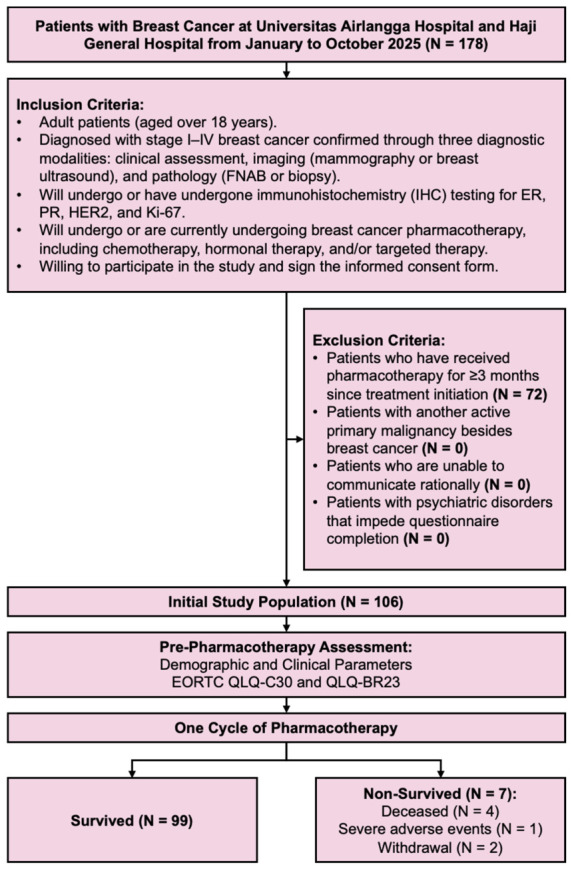
Flowchart of Study Design.

Adult women aged ≥ 18 years were eligible for inclusion if they had a confirmed diagnosis of stage I–IV breast cancer, established through concordant clinical evaluation, breast imaging (mammography or ultrasonography), and histopathological verification using fine-needle aspiration cytology or core needle biopsy. Additional inclusion criteria required the availability or planned assessment of immunohistochemistry (IHC) markers—including estrogen receptor (ER), progesterone receptor (PR), HER2, and Ki-67—and initiation or continuation of systemic pharmacotherapy, including chemotherapy, endocrine therapy, or targeted therapy. Exclusion criteria comprised prior receipt of systemic therapy for ≥3 months, presence of another active primary malignancy, cognitive or communication impairment that prevented reliable questionnaire completion, and psychiatric disorders interfering with the validity of PRO assessment. Patients meeting these criteria were consecutively recruited at both centers during the study period (Figure 1).

### 2.4. Data Collection and Baseline Assessment

At enrollment, trained research staff collected demographic and clinical characteristics, including age, education level, employment status, marital status, comorbidities, tumor stage, metastatic sites, histopathology, tumor grade, and IHC subtype. Treatment-related data included planned or ongoing pharmacotherapy regimen, intent (neoadjuvant, adjuvant, or palliative), and concurrent modalities such as surgery or radiotherapy, abstracted from medical records. Baseline PROs were assessed prior to initiation of the first pharmacotherapy cycle using the validated Indonesian versions of the EORTC QLQ-C30 and EORTC QLQ-BR23 questionnaires. Patients completed the instruments independently, with assistance provided only for illiteracy or visual limitations, without influencing item interpretation. Scores were transformed according to EORTC scoring manuals to generate standardized 0–100 scales for functional domains, symptom domains, and global health status.

### 2.5. Outcome Definition

The primary outcome was survival of the first cycle of systemic pharmacotherapy, defined as the absence of the combined event of death from any cause before completion of the first cycle, or treatment discontinuation due to adverse effects, including patient-initiated withdrawal based on severe symptoms or intolerance. Patients who completed the first treatment cycle without meeting either criterion were classified as survivors of the early pharmacotherapy phase.

### 2.6. Statistical Analysis

Baseline characteristics were summarized using descriptive statistics. Continuous variables were expressed as means ± standard deviations or medians with interquartile ranges (IQRs), depending on data distribution. Normality of continuous variables was assessed prior to analysis. Given the non-normal distribution of QoL data, comparisons of baseline PROs between survivors and non-survivors were performed using the Mann–Whitney U test. Categorical variables were presented as frequencies and percentages and compared using the Chi-square test of independence. To evaluate the discriminative performance of baseline PRO domains in relation to survival during the first cycle of pharmacotherapy, receiver operating characteristic (ROC) curve analyses were conducted. The area under the curve (AUC) was calculated for each domain to quantify discriminative accuracy, with corresponding *p*-values used to assess statistical significance. An AUC value of 0.5 indicated no discriminative ability, whereas higher values reflected increasing discriminatory performance. For global health status (QL2), additional diagnostic performance analyses were performed across multiple threshold values. Sensitivity and the false positive rate (1 − specificity) were calculated for each cut-off point. The Youden index (J) was derived using the formula J = sensitivity − (1 − specificity), to identify the threshold that provided the optimal balance between sensitivity and specificity. Higher Youden index values indicated better overall discriminative performance. All statistical analyses were conducted using IBM SPSS version 27 (IBM Corp., Armonk, NY, USA), with statistical significance defined as a two-sided *p*-value < 0.05.

## 3. Results

### 3.1. Baseline Demographic and Clinical Characteristics

A total of 106 women with breast cancer were enrolled, comprising 99 survivors and 7 non-survivors during the first cycle of pharmacotherapy. All participants were female. The mean age of survivors was 52.1 ± 9.5 years, slightly higher than the 49.1 ± 12.5 years observed among non-survivors, though age distributions were broadly comparable (Table 1). Educational attainment differed modestly between groups: among survivors, 33.3% had completed high school and 38.4% held university-level education, whereas non-survivors were more evenly distributed across elementary, secondary, and high school levels, with only one patient (14.3%) having university education. Employment status also varied, with 64.6% of survivors being unemployed or homemakers compared with 34.0% among non-survivors, whereas employed individuals represented 35.4% and 66.0% of the respective groups. Marital status was predominantly married among survivors (84.8%) and uniformly married in the non-survivor group (100%). Comorbidities were common in both groups, present in 59.6% of survivors and 57.1% of non-survivors. Hypertension and cardiovascular disease were the most frequently documented comorbid conditions, with similar proportions across groups, although lung and pleural diseases were reported only among survivors. Regarding prior breast cancer treatment, 69.7% of survivors and 71.4% of non-survivors had received previous oncologic therapy. Most survivors had undergone surgery (56 patients) and pharmacotherapy (44 patients), while smaller proportions had received radiotherapy (3 patients). Non-survivors exhibited similar patterns, with 4 having undergone surgery, 5 having received systemic therapy, and 1 having previously completed radiotherapy.

### 3.2. Tumor Characteristics and Treatment Profiles

Tumor-related characteristics differed between survivors (n = 99) and non-survivors (n = 7) at baseline (Table 2). Among survivors, the majority had locally advanced disease (60.6%), followed by metastatic disease (25.3%) and early-stage breast cancer (14.1%). In contrast, non-survivors were predominantly diagnosed with metastatic breast cancer (71.4%), while the remaining patients (28.6%) had locally advanced tumors; no cases of early-stage disease were documented in this group. Patterns of metastatic involvement also varied. Among survivors, lung–pleural metastases were the most common (13 patients), followed by liver (8 patients), bone (7 patients), and brain metastases (1 patient). Non-survivors exhibited lung–pleural metastases in 3 patients, bone metastases in 3 patients, and liver metastasis in 1 patient; none had brain involvement. Histopathological subtypes were dominated by invasive ductal carcinoma (IDC) in both groups, accounting for 84.8% of cases among survivors and 71.4% among non-survivors. Mixed IDC/invasive lobular carcinoma (ILC) was observed in 7.1% and 14.3% of survivors and non-survivors, respectively. Rare histologies and unknown subtypes were infrequent in both cohorts. Tumor grading showed a broad distribution among survivors, with Grade III tumors constituting the largest category (39.4%), followed by Grade II (36.4%) and Grade I (10.1%). Non-survivors predominantly had Grade III (57.1%) and Grade II (28.6%) tumors. IHC profiles were heterogeneous across groups. Among survivors, the most frequent subtypes were Luminal B HER2− (27.3%), Luminal B HER2+ (19.2%), and HER2-enriched (16.2%), followed by Luminal A (9.1%), HER2-low (6.1%), and triple-negative breast cancer (7.1%). Non-survivors were distributed across Luminal A, Luminal B HER2−, Luminal B HER2+, HER2-low, and triple-negative categories (all 14.3%), with no cases of HER2-enriched disease and two cases (28.6%) with unknown status. 

Pharmacotherapy intent differed between groups. Among survivors, treatment was administered in neoadjuvant (35.4%), adjuvant (39.4%), or palliative settings (25.3%). Non-survivors received therapy predominantly in the palliative setting (71.4%), with only one patient each receiving neoadjuvant or adjuvant therapy. The pharmacotherapy regimens utilized in both groups were diverse, reflecting standard anthracycline-, taxane-, and platinum-based combinations. Survivors most frequently received docetaxel–carboplatin (18 patients), doxorubicin–cyclophosphamide (17 patients), epirubicin–docetaxel–trastuzumab (11 patients), and paclitaxel–carboplatin (8 patients). In contrast, regimens among non-survivors were fewer in number, with epirubicin–docetaxel–trastuzumab (2 patients) and carboplatin–gemcitabine (2 patients) being the most frequently administered combinations. These tumor characteristics and treatment distributions are summarized in Table 2.

### 3.3. Baseline Pre-Pharmacotherapy Quality-of-Life Scores

Baseline patient-reported quality-of-life (QoL) scores, measured using the EORTC QLQ-C30 and QLQ-BR23 instruments, demonstrated notable differences between survivors (n = 99) and non-survivors (n = 7) at the start of pharmacotherapy. Survivors reported substantially higher global health status/QoL, with a median score of 83.3 [IQR 66.7–91.7], compared with 41.7 [25.0–50.0] among non-survivors (*p* < 0.001). On functional scales, survivors also had higher physical functioning (93.3 vs. 66.7; *p* = 0.027), role functioning (100.0 vs. 66.7; *p* = 0.009), emotional functioning (83.3 vs. 41.7; *p* = 0.002), and social functioning (100.0 vs. 66.7; *p* < 0.001). Cognitive functioning scores were high in both groups, with no significant difference (*p* = 0.362). Symptom burden was higher among non-survivors across several QLQ-C30 domains. Median pain scores were greater in non-survivors (66.7 vs. 16.7; *p* = 0.008), along with higher scores for appetite loss (33.3 vs. 0.0; *p* = 0.010), constipation (33.3 vs. 0.0; *p* = 0.010), and financial difficulty (33.3 vs. 0.0; *p* = 0.047). Differences in fatigue, dyspnea, insomnia, and nausea/vomiting did not reach statistical significance. On the QLQ-BR23 functional scales, future perspective was lower among non-survivors (33.3 vs. 66.7; *p* = 0.014), while body image, sexual functioning, and sexual enjoyment showed no significant group differences. For BR23 symptom scales, non-survivors reported higher systemic therapy side effects (23.8 vs. 9.5; *p* = 0.037) and arm symptoms (44.4 vs. 11.1; *p* = 0.050). Scores for breast symptoms and hair-loss–related distress were comparable between groups. A complete summary of QoL differences is presented in Table 3.

### 3.4. Receiver Operating Characteristic (ROC)-Based Evaluation of QoL Measures in Predicting Early Treatment Outcomes

**Figure 2:**
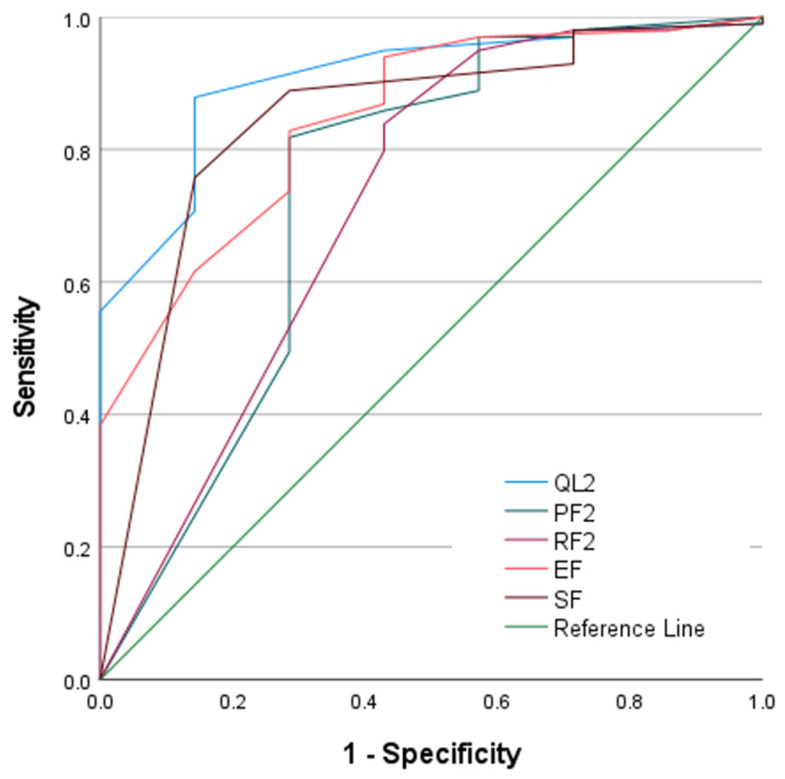
ROC Curves of EORTC QLQ-C30 Global Health Status and Functional Domains for Predicting First-Cycle Pharmacotherapy Survival.

ROC analyses of baseline EORTC QLQ-C30 global health status and functional domains demonstrated varying levels of discriminative performance for predicting survival during the first cycle of pharmacotherapy (Figure 2). The QL2 domain showed the most pronounced separation between survivors and non-survivors, with the ROC curve consistently positioned far above the reference line across threshold values. Among the functional domains, emotional functioning (EF) and social functioning (SF) also displayed strong discriminatory patterns, with curves closely approximating that of global health status. Physical functioning (PF2) and role functioning (RF2) exhibited more moderate discrimination, with ROC curves deviating from the reference line to a lesser extent. Overall, the graphical patterns indicate that baseline global and functional QoL domains provide measurable ability to distinguish early pharmacotherapy outcomes. Quantitative assessment of these ROC findings is presented in Table 4. QL2 achieved the highest discriminative accuracy, with an AUC of 0.907 (*p* < 0.001). EF and SF also demonstrated strong performance, with AUC values of 0.846 (*p* = 0.002) and 0.843 (*p* = 0.003), respectively. PF2 showed moderate discrimination, with an AUC of 0.737 (*p* = 0.037). RF2 yielded an AUC of 0.718, which did not reach statistical significance (*p* = 0.055).

**Figure 3:**
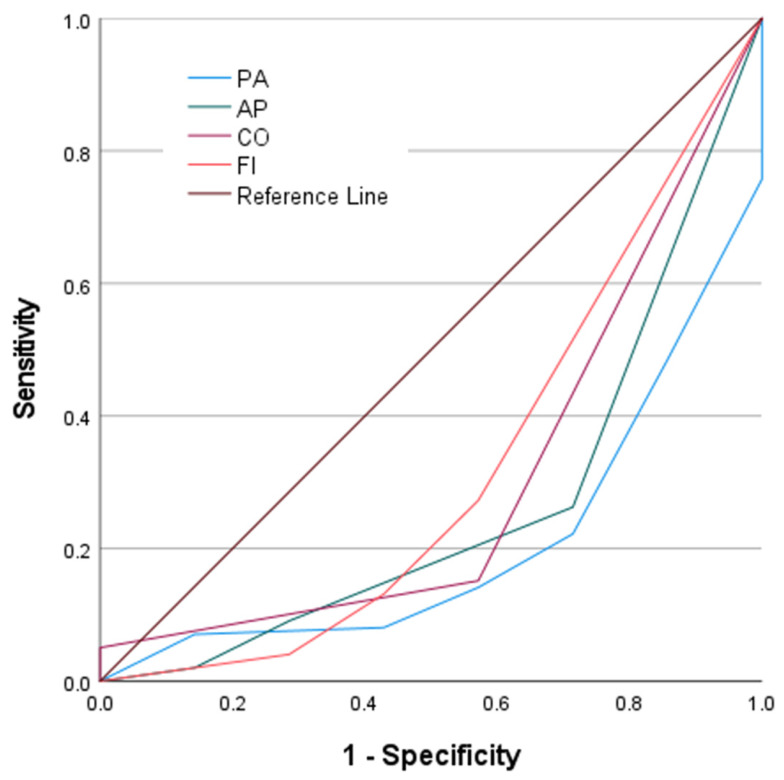
ROC Curves of EORTC QLQ-C30 Symptom Domains for Predicting First-Cycle Pharmacotherapy Survival.

ROC analyses of the EORTC QLQ-C30 symptom domains demonstrated variable and generally lower discriminative performance for predicting survival during the first cycle of pharmacotherapy (Figure 3). The ROC curves for pain (PA) and appetite loss (AP) showed the most noticeable deviation from the reference line among the symptom domains, although their trajectories were oriented below the diagonal, indicating an inverse direction of discrimination. In contrast, constipation (CO) and financial difficulty (FI) exhibited ROC curves that remained closer to the reference line, suggesting limited ability to distinguish between survivors and non-survivors. Overall, the graphical patterns indicate that symptom domains provide weaker and less consistent separation compared with global health status and functional scales. The quantitative results of these analyses are presented in Table 5. PA demonstrated an AUC of 0.208 (*p* = 0.010), while AP yielded an AUC of 0.266 (*p* = 0.039), both reaching statistical significance despite low AUC values. CO had an AUC of 0.304 (*p* = 0.085), and FI showed an AUC of 0.320 (*p* = 0.112), neither of which were statistically significant. These findings indicate that the evaluated symptom domains have limited discriminative accuracy for early pharmacotherapy survival, with only pain and appetite loss demonstrating statistically significant but low AUC values.

**Figure 4:**
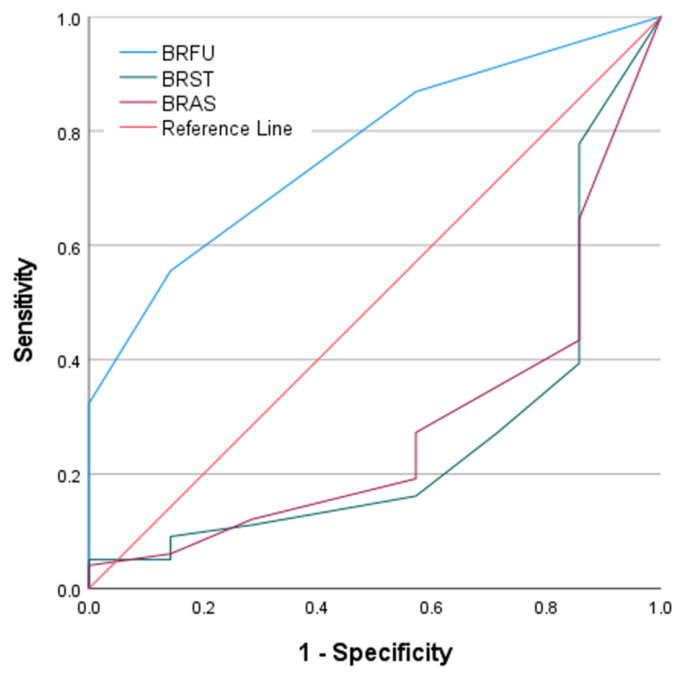
ROC Curves of EORTC QLQ-BR23 Domains for Predicting First-Cycle Pharmacotherapy Survival.

Next, ROC analyses of the EORTC QLQ-BR23 domains demonstrated heterogeneous discriminative performance for predicting survival during the first cycle of pharmacotherapy (Figure 4). The ROC curve for future perspective (BRFU) showed the most distinct separation from the reference line, indicating a relatively stronger ability to differentiate between survivors and non-survivors. In contrast, the curves for systemic therapy side effects (BRST) and arm symptoms (BRAS) were positioned closer to or below the diagonal reference line, reflecting weaker and less consistent discrimination, with an inverse orientation observed for BRST. The corresponding quantitative results are presented in Table 6. BRFU demonstrated moderate discriminative accuracy, with an AUC of 0.768 (*p* = 0.018). BRST yielded an AUC of 0.266 (*p* = 0.039), indicating statistically significant but low discriminative performance with inverse direction. BRAS showed an AUC of 0.284 (*p* = 0.056), which did not reach statistical significance.

Next, the discriminative performance of QL2 across multiple threshold values further demonstrated a clear trade-off between sensitivity and the false positive rate (1 − specificity) in distinguishing early pharmacotherapy survival (Table 7). At the lowest cut-off values (e.g., −1.0 and 12.5), sensitivity was maximal (1.000 and 0.990, respectively), but this was accompanied by the highest false positive rates (1.000), resulting in low or negative Youden indices (0.000 and −0.010), indicating poor overall discriminative utility. As the threshold increased, sensitivity gradually declined while the false positive rate decreased, reflecting improved specificity. Intermediate cut-off values, particularly in the range of approximately 29.2 to 45.8, demonstrated moderate Youden indices (0.256–0.520), indicating progressively improved balance between sensitivity and specificity. The highest Youden index was observed at a QL2 cut-off of approximately 54.2 (J = 0.736), corresponding to a sensitivity of 0.879 and a low false positive rate of 0.143 (specificity 0.857), representing the optimal balance of classification performance. At higher thresholds (≥62.5), the Youden index declined despite further reductions in false positive rate, due to a more pronounced loss of sensitivity. At the highest cut-off values (≥79.2), the false positive rate approached zero, reflecting maximal specificity but substantially reduced sensitivity, thereby limiting overall discriminative performance. Collectively, these findings indicate that QL2 provides the most effective discrimination at intermediate-to-high threshold values, with an optimal cut-off around 50–55, supporting its role as a clinically meaningful marker for early risk stratification rather than a purely sensitivity-driven screening tool.

## 4. Discussion

### 4.1. Interpretation of Baseline Demographic and Clinical Characteristics

The baseline demographic and clinical characteristics of the cohort provide important context for understanding early pharmacotherapy outcomes and the prognostic value of PROs. In this study, survivors and non-survivors exhibited broadly similar age distributions, with mean ages in the late 40s to early 50s, consistent with the relatively young age of breast cancer onset commonly reported in Southeast Asian populations [15]. This age pattern suggests that chronological age alone was not a strong determinant of short-term pharmacotherapy survival, aligning with emerging evidence that functional reserve, symptom burden, and psychosocial factors may supersede age as early prognostic markers. Differences in educational attainment and employment status between survivors and non-survivors highlight potential socioeconomic influences on treatment tolerance and continuity. A larger proportion of survivors held higher levels of education and formal employment, whereas non-survivors were more evenly distributed across lower educational strata. Education has been shown to correlate with improved health literacy, treatment adherence, and symptom reporting—factors that may influence both the ability to endure pharmacotherapy and the accuracy of baseline PRO assessments [16,17]. The uniformly married status of non-survivors may also reflect differing social support structures, which can affect coping mechanisms and engagement with symptom management during early treatment [18].

Comorbidity profiles revealed that more than half of patients in both groups had pre-existing medical conditions, most commonly hypertension and cardiovascular diseases. This suggests that comorbidity severity or functional impact, rather than presence alone, may be more relevant in shaping tolerance to pharmacotherapy [19]. These findings underscore the importance of complementing traditional clinical assessments with PROs, which are better suited to capture the subjective impact of comorbid illnesses on daily functioning. Prior oncologic treatments, particularly surgery and systemic therapy, were common among both survivors and non-survivors, indicating that treatment exposure alone did not predict early-cycle survival. However, the relatively high proportion of survivors who had previously undergone surgery or adjuvant therapy may indicate greater engagement with cancer care pathways or earlier disease presentation. Importantly, the similarity of these clinical variables between groups reinforces the central hypothesis of this study: traditional demographic and clinical characteristics, while clinically informative, may inadequately identify patients at risk of early treatment failure, especially within the compressed timeframe of the first pharmacotherapy cycle.

### 4.2. Tumor Biology and Treatment Patterns in Relation to Early-Cycle Outcomes

The tumor characteristics presented in Table 2 highlight substantial clinical differences between survivors and non-survivors during the first pharmacotherapy cycle, offering important context for understanding the prognostic performance of baseline PROs. As expected, disease stage at presentation demonstrated a clear separation between groups, with non-survivors exhibiting a markedly higher proportion of metastatic breast cancer (71.4%) compared with survivors (25.3%). This pattern aligns with established evidence that advanced disease confers greater biological aggressiveness, higher symptom burden, and reduced tolerance to systemic therapy [20,21]. However, while stage remains a major determinant of long-term survival, its ability to predict early-cycle treatment failure has been shown to be limited, underscoring the need for complementary prognostic tools such as PROs that capture functional reserve and symptomatic vulnerability more directly. Patterns of metastatic spread were broadly consistent with global metastatic breast cancer cohorts, with lung–pleural, liver, and bone metastases representing the most common sites [22]. The slightly greater clustering of visceral metastases among non-survivors reflects a clinical phenotype associated with more severe systemic symptoms, yet these clinical markers still do not fully capture patient-specific impact on daily functioning—an aspect more accurately measured by PRO instruments such as the EORTC QLQ-C30 and BR23. Similarly, while IDC predominated in both groups, and tumor grade tended to be higher among non-survivors, these pathological features offer limited insight into the physiological or psychosocial factors that influence early discontinuation due to toxicity. IHC subtypes were heterogeneous across the cohort, mirroring the molecular diversity of breast cancer. Although non-survivors were distributed across luminal, HER2-low, and triple-negative phenotypes without a clear dominant pattern, the absence of HER2-enriched tumors contrasts with survivors, among whom HER2-enriched disease accounted for 16.2% of cases. Importantly, molecular subtype did not exhibit a distinct or consistent separation between groups, further reinforcing the limitations of relying solely on biological tumor profiles to anticipate early treatment intolerance. In contemporary oncology, IHC subtype is a strong predictor of long-term prognosis and treatment response, yet it may be less informative in capturing acute vulnerabilities during the initial treatment cycle—again emphasizing the value of PRO-derived functional and symptom scores [23,24].

Differences in treatment intent were more pronounced: while survivors were distributed across neoadjuvant, adjuvant, and palliative settings, the majority of non-survivors were receiving palliative therapy (71.4%). Although this reflects underlying disease burden, it also highlights the heterogeneity in treatment objectives across the cohort. Despite this, the diversity of chemotherapy regimens administered suggests that treatment type alone did not account for early-cycle mortality or discontinuation. Survivors received a wider range of anthracycline-, taxane-, and trastuzumab-based regimens, while non-survivors predominantly received a smaller subset of combinations. Notably, regimen complexity did not correspond directly with survival outcomes, indicating that clinical tolerance to therapy may depend more on pre-treatment functional status and symptom profiles than on regimen selection. Taken together, the findings from Table 2 underscore a critical insight: traditional tumor characteristics—including stage, metastatic pattern, histopathology, grade, and molecular subtype—while essential for long-term prognostication, do not fully explain early pharmacotherapy survival outcomes. The limited predictive separation demonstrated by these clinical and pathological variables highlights the potential for PROs to substantially augment baseline risk stratification.

### 4.3. Baseline Functional and Symptom Burden: Implications for Early Outcomes

QoL findings presented in Table 3 reveal the strongest and most clinically meaningful distinctions between survivors and non-survivors during the first cycle of pharmacotherapy. These results underscore the central premise of this study: baseline PROs—captured through the EORTC QLQ-C30 and QLQ-BR23 instruments—offer powerful, multidimensional insights into patient vulnerability that are not easily captured by traditional clinical or tumor-based parameters. The markedly lower global health status/QoL among non-survivors (median 41.7 vs. 83.3; *p* < 0.001) highlights Global Health as a sensitive, composite indicator reflecting physical, psychological, and functional reserves before treatment initiation. This aligns with previous studies demonstrating that global QoL is among the strongest independent predictors of survival across multiple cancer types [25,26,27]. Functional domains demonstrated a similarly consistent pattern. Non-survivors scored significantly lower on physical, role, emotional, and social functioning, suggesting profound impairments across daily activity, coping capacity, and social engagement—all factors known to influence treatment adherence and tolerance. The absence of significant differences in cognitive functioning indicates that physical rather than cognitive limitations may play a more determinative role in early-cycle outcomes. Importantly, these functional impairments likely reflect both disease burden and underlying physiologic frailty, collectively contributing to reduced resilience against treatment-related toxicity.

The symptom scales further reinforce the prognostic significance of PROs. Non-survivors exhibited substantially higher pain, appetite loss, and constipation, alongside greater systemic therapy side effects and arm symptoms. These symptoms not only reflect underlying tumor biology, especially in advanced disease, but also represent early warning signals of treatment intolerance. Elevated pain and appetite loss, in particular, are well-established markers of catabolic stress, poor nutritional reserve, and diminished performance status—all of which can accelerate clinical deterioration during chemotherapy. The significantly higher financial difficulty score among non-survivors provides additional insight into psychosocial vulnerability, which may influence access to supportive care, nutrition, and early symptom management. The breast cancer–specific BR23 scales provide additional insight. The significantly lower future perspective score among non-survivors suggests heightened psychological distress and diminished treatment optimism, factors that have previously been associated with early discontinuation and lower adherence. In contrast, body image and sexual function scores showed no group differences, indicating that survival-relevant QoL distinctions were concentrated in domains more directly associated with physical function and systemic symptoms. Taken together, these findings demonstrate that PROs outperform traditional clinical characteristics in discriminating early survivors from non-survivors, providing a comprehensive and patient-centered risk profile. The clear and consistent separation across multiple QoL domains underscores the clinical value of integrating EORTC instruments into baseline assessment, particularly for identifying high-risk patients before the first pharmacotherapy cycle—when the opportunity for intervention is greatest [10,28].

### 4.4. Discriminative Performance of Baseline PRO Domains for Early Pharmacotherapy Outcomes

The ROC analyses presented in Section 3.4 provide important quantitative confirmation of the strong prognostic signal observed in baseline PRO comparisons, demonstrating that selected EORTC QLQ-C30 and QLQ-BR23 domains possess measurable discriminative accuracy for early pharmacotherapy survival. Notably, QL2 exhibited excellent performance (AUC 0.907), while EF and SF also showed strong discrimination (AUCs 0.846 and 0.843, respectively). These findings are highly consistent with existing literature, which has repeatedly identified global QoL as one of the most robust predictors of survival across cancer populations [10,29]. Large-scale analyses, including pooled data from multiple clinical trials, have demonstrated that baseline QLQ-C30 global health scores independently predict overall survival, often outperforming traditional clinical variables such as tumor stage or performance status [10]. The present findings extend this evidence into a much earlier and clinically critical timeframe—the first cycle of pharmacotherapy—suggesting that the prognostic value of global QoL is not limited to long-term outcomes but is already strongly operative at treatment initiation. The strong performance of EF and SF domains is also aligned with emerging evidence highlighting the role of psychosocial health in cancer outcomes [7,30]. While earlier studies have traditionally emphasized physical functioning as the dominant prognostic domain, more recent work suggests that emotional distress, social isolation, and impaired coping mechanisms significantly influence treatment adherence, symptom perception, and physiologic resilience [31,32,33]. The relatively high AUCs observed for EF and SF in this study reinforce the concept that psychosocial domains are not merely secondary correlates of disease burden, but integral components of early treatment tolerance. This is particularly relevant in LMIC settings, where social support structures and psychological resources may vary widely and directly impact patients’ ability to navigate early treatment-related challenges. PF2 and RF2 demonstrated moderate discriminative ability, which is broadly consistent with prior literature but somewhat less pronounced than expected. In many studies, physical functioning has been one of the strongest predictors of survival, often closely mirroring clinician-assessed performance status (e.g., ECOG) [34,35]. The comparatively lower AUC observed here (0.737) may reflect the relatively homogeneous baseline physical status of patients deemed fit to initiate pharmacotherapy, thereby reducing variability and limiting discriminatory power. Additionally, because this study focuses specifically on very early outcomes, it is plausible that broader constructs such as global health and emotional resilience capture early vulnerability more effectively than isolated measures of physical capacity.

In contrast, the symptom domains of the QLQ-C30 demonstrated limited and, in some cases, inverse discriminative performance. PA and AP reached statistical significance but exhibited low AUC values (<0.3), indicating poor classification accuracy when interpreted in the conventional direction. This apparent paradox is likely explained by the directional properties of symptom scales, where higher scores indicate worse symptom burden. When not inverted, ROC analyses may yield AUC values below 0.5 despite meaningful associations. Similar patterns have been reported in prior studies, where symptom scales—although prognostically relevant—often demonstrate weaker and less stable discriminative performance compared with functional domains. This suggests that while individual symptoms reflect specific aspects of disease burden, they may lack the integrative capacity required for robust outcome prediction when considered in isolation [36]. Furthermore, symptom reporting may be influenced by subjective thresholds, cultural factors, and reporting variability, which can attenuate their predictive consistency [37,38]. Findings from the QLQ-BR23 module further support the differential prognostic value of functional versus symptom domains. BRFU demonstrated moderate discriminative ability (AUC 0.768), highlighting the importance of psychological outlook and illness perception in early treatment outcomes. This aligns with psycho-oncology literature suggesting that patients’ expectations, optimism, and perceived future orientation are associated with adherence, engagement in care, and even biological stress responses [39]. In contrast, BR23 symptom domains such as systemic therapy side effects and arm symptoms showed low or non-significant AUCs, again reflecting limited discriminatory performance. These findings are consistent with prior reports indicating that breast cancer–specific symptom scales, while clinically informative, are less predictive of survival outcomes than broader QoL constructs.

Further insight into the discriminative performance of QL2 is provided by threshold-based analysis incorporating sensitivity, false positive rate (1 − specificity), and the Youden index. While very low QL2 cut-off values demonstrated maximal sensitivity, these were accompanied by extremely high false positive rates, resulting in poor overall discriminatory performance. In contrast, intermediate-to-higher thresholds showed a more favorable balance between correctly identifying patients at risk and minimizing misclassification. Notably, the highest Youden index was observed at a QL2 cut-off of approximately 54, corresponding to high sensitivity (0.879) and low false positive rate (0.143), indicating optimal combined performance. This finding suggests that the prognostic utility of QL2 is not driven by extreme values alone but reflects a clinically meaningful threshold range where both sensitivity and specificity are reasonably preserved. Importantly, this pattern highlights that optimal discrimination arises from a balance between detection and exclusion, rather than prioritizing sensitivity alone, reinforcing the value of ROC-based threshold analysis in translating PRO measures into clinically actionable tools. When viewed collectively, the ROC and threshold-based analyses reinforce a key conceptual distinction in PRO research: composite and functional domains (e.g., global health, emotional functioning) provide superior prognostic discrimination compared with isolated symptom measures [40]. This pattern has been consistently observed across multiple cancer types and study designs. The present findings extend this evidence by demonstrating that such discriminative performance is already evident at the earliest phase of systemic therapy. Importantly, the identification of an optimal QL2 threshold in the intermediate-to-high range further supports the clinical applicability of baseline PROs, suggesting that global health status may serve not only as a continuous risk marker but also as a practical tool for early risk stratification. This is particularly relevant in the context of early-cycle outcomes, where timely identification of vulnerable patients can directly inform supportive care interventions and treatment planning [41,42,43]. The magnitude of AUC observed for QL2 in this study is notably high, likely reflecting the close relationship between early pharmacotherapy outcomes and baseline functional reserve. Together, these findings underscore the potential of PROs to bridge the gap between subjective patient experience and objective clinical decision-making, particularly in resource-limited settings where rapid, low-cost prognostic tools are critically needed.

## 5. Clinical Implications

The findings of this study demonstrate that baseline PROs offer substantial prognostic value for identifying breast cancer patients at risk of early mortality or treatment discontinuation during the first cycle of systemic therapy. This has several important clinical implications for oncology practice, particularly in resource-constrained settings. First, the pronounced differences in global health status, functional capacity, and symptom burden between survivors and non-survivors highlight the need for routine integration of validated PRO instruments—such as the EORTC QLQ-C30 and QLQ-BR23—into baseline assessment [28]. Incorporating these measures provides a more complete representation of a patient’s physiological reserve, functional limitations, and psychosocial vulnerabilities than conventional clinical or tumor characteristics alone [44]. This is especially relevant when clinicians must determine whether a patient is sufficiently fit to initiate therapy, select an appropriate regimen, or anticipate early complications.

Second, the ability of PROs to identify high-risk patients prior to treatment initiation offers a critical opportunity for proactive supportive care interventions. Patients with poor baseline functioning, high pain scores, significant appetite loss, or elevated financial distress may benefit from prehabilitation strategies, nutritional optimization, pain control, psychosocial counseling, and financial navigation support [45,46]. Early deployment of these interventions may improve treatment tolerance, reduce the risk of early discontinuation, and ultimately enhance overall outcomes. Importantly, such supportive strategies can be implemented without altering oncologic treatment intent, making PRO-guided care both feasible and scalable across secondary referral hospitals. The ROC analyses further refine these findings by demonstrating that not all PRO domains contribute equally to prognostic discrimination. Global health status, emotional functioning, and social functioning exhibited the highest discriminative accuracy, supporting their prioritization as key indicators for early risk stratification. These results suggest a tiered implementation approach, in which high-performing domains may be used for rapid screening, particularly in resource-limited settings where comprehensive PRO assessment may not be feasible. In contrast, symptom domains showed limited discriminative performance, indicating that while they remain essential for clinical management, they may be less suitable as standalone predictors of early treatment survival and should be interpreted alongside broader functional measures. The strong performance of selected PRO domains also supports their integration into clinical decision-support frameworks to guide treatment intensity, monitoring strategies, and early supportive care interventions. Importantly, these findings demonstrate that the prognostic value of PROs is already evident at treatment initiation, underscoring their role as immediate tools for optimizing early pharmacotherapy outcomes.

Third, the findings support the use of PROs as decision-support tools for tailoring treatment intensity. For patients presenting with severely impaired QoL or high symptom burden, clinicians may consider initiating therapy with modified dosing, sequential rather than combination regimens, or enhanced toxicity monitoring. For others with preserved functional status, standard treatment intensity may be maintained with greater confidence. By guiding individualized treatment selection, PROs can contribute to more precise and patient-centered pharmacotherapy planning. Finally, routine PRO assessment has important implications for health system strengthening [47]. In settings such as Indonesia, where oncologist workloads are high and supportive oncology resources are limited, PROs offer a scalable means of enhancing patient triage and monitoring without increasing the burden on clinical staff. Digital or paper-based PRO collection can be implemented efficiently, enabling rapid identification of patients who require urgent evaluation, psychosocial support, or early toxicity management [48]. In this way, PROs can function as an early-warning system that improves overall quality of care, enhances patient-provider communication, and ensures more equitable access to supportive services. Collectively, these implications underscore the strong potential of baseline PROs to transform clinical practice by offering an accessible, patient-centered, and prognostically meaningful approach to risk stratification and early supportive care in breast cancer pharmacotherapy. Integrating PROs into routine workflows could significantly improve treatment continuity, patient safety, and early outcomes—particularly within secondary referral hospitals and LMIC oncology settings where the need for efficient, high-impact tools is greatest.

## 6. Study Limitations and Future Directions

This study has several limitations that should be acknowledged when interpreting its findings. First, although prospectively conducted, the sample size—particularly the small number of non-survivors (n = 7)—limits the statistical power to detect more nuanced associations between specific clinical variables and early treatment outcomes. The low event rate also restricts the ability to perform multivariable analyses that could more robustly disentangle the independent contributions of tumor characteristics, treatment intent, and baseline PROs. Larger multicenter cohorts will be essential to validate the prognostic thresholds suggested by this study and improve the generalizability of its findings. Second, the study was conducted in two secondary referral hospitals in Indonesia, which share similar referral patterns and resource constraints. While this strengthens internal consistency, it may limit external validity for settings with different healthcare infrastructure, socioeconomic profiles, or supportive oncology services. Moreover, because the study focused on survival and treatment completion during the first cycle of pharmacotherapy, long-term survival outcomes, cumulative toxicity patterns, and dynamic changes in PROs were not captured. PRO trajectories over time—rather than baseline scores alone—may provide richer prognostic insight and are an important avenue for future research. Third, although the EORTC QLQ-C30 and BR23 are well-validated, PROs remain subjective and may be influenced by cultural factors, health literacy, psychological resilience, and social desirability bias. While trained research staff minimized measurement inconsistencies, the potential for reporting bias cannot be fully eliminated. Additionally, the absence of standardized supportive care pathways across both hospitals may have contributed to variability in symptom control, adherence, and early discontinuation, potentially affecting the relationship between baseline PROs and outcomes.

Despite these limitations, this study provides important opportunities for future investigation. Larger, multicenter prospective cohorts across diverse healthcare settings are needed to refine PRO-based risk models and establish clinically actionable cut-offs for early treatment failure. Integrating serial PRO assessments can help characterize how fluctuations in symptom burden or functional decline predict toxicity, adherence, and overall survival. Further, combining PROs with objective physiologic markers—such as nutritional status, inflammatory biomarkers, or performance metrics—may yield composite prognostic indices with enhanced predictive accuracy. Finally, emerging digital health tools, including mobile-based PRO collection platforms and automated triage algorithms, represent promising avenues to operationalize PRO-driven risk stratification at scale [48]. Future research should explore the feasibility, acceptability, and clinical impact of digital PRO monitoring systems in LMICs, where resource constraints heighten the need for low-cost, high-yield prognostic tools. By expanding on the foundational insights from this study, future work can advance the integration of PROs into routine oncology care and contribute to more personalized, equitable, and effective breast cancer treatment delivery.

## 7. Conclusions

In this prospective cohort study, baseline patient-reported outcomes measured using the EORTC QLQ-C30 and QLQ-BR23 emerged as strong prognostic indicators of early pharmacotherapy survival in women with breast cancer, outperforming traditional demographic, clinical, and tumor-based indicators. Markedly lower global health, impaired functional status, and higher symptom burden consistently characterized patients who died or discontinued treatment during the first cycle. These findings highlight the critical value of integrating PRO assessments into routine pre-treatment evaluation to identify high-risk patients, guide individualized supportive care, and enhance treatment readiness—particularly within resource-limited secondary referral hospital settings. Incorporating PROs into initial decision-making pathways may improve early treatment continuity, patient safety, and overall quality of oncology care.

## Figures and Tables

**Table 1:** Baseline demographic and clinical characteristics of survived vs. non-survived patients.

Parameter	Survived (N = 99)	Non-Survived (N = 7)	*p*-Value *^#^*
**Sex (n; %)**			
Female	99 (100.0%)	7 (100.0%)	-
**Age in years (mean ± SD)**	52.11 ± 9.5	49.14 ± 12.5	0.435
**Educational background (n; %)**			
No formal education	2 (2.0%)	0 (0.0%)	0.707
Elementary school	20 (20.2%)	2 (28.6%)	0.598
Secondary school	6 (6.1%)	2 (28.6%)	0.031 *
High school	33 (33.3%)	2 (28.6%)	0.799
University-level education	38 (38.4%)	1 (14.3%)	0.204
**Employment status (n; %)**			
Unemployed/housewife	64 (64.6%)	6 (34.0%)	0.108
Employed	35 (35.4%)	1 (66.0%)	0.108
**Marital status (n; %)**			
Single/unmarried	8 (8.1%)	0 (0.0%)	0.436
Married	84 (84.8%)	7 (100.0%)	0.268
Widow	7 (7.1%)	0 (0.0%)	0.468
**Comorbidities (n; %)**			
**Absent**	40 (40.4%)	3 (42.9%)	0.897
**Present:**	59 (59.6%)	4 (57.1%)	0.897
Hypertension (n)	30	2	-
Diabetes mellitus (n)	10	2	-
Cardiovascular diseases (n)	13	3	-
Lung and pleural diseases (n)	7	0	-
Others (n)	37	0	-
**Treatment history for breast cancer (n; %)**			
**Absent**	30 (30.3%)	2 (28.6%)	0.925
**Present**	69 (69.7%)	5 (71.4%)	0.925
Surgery (n)	56	4	-
Pharmacotherapy (n)	44	5	-
Radiotherapy (n)	3	1	-

^#^ Comparisons between proportions were performed using the Chi-Square Test of Independence. * statistically significant at *p* < 0.05. SD = standard deviation; n = number of patients; % = percentage.

**Table 2:** Breast cancer profile of survived vs. non-survived patients.

Parameter	Survived (N = 99)	Non-Survived (N = 7)	*p*-Value *^#^*
**Breast cancer stage (n; %)**			
Early	14 (14.1%)	0 (0.0%)	0.289
Locally advanced	60 (60.6%)	2 (28.6%)	0.098
Metastatic	25 (25.3%)	5 (71.4%)	0.009 *
**Metastatic site (n)**			
Brain	1	0	-
Lung—pleura	13	3	-
Liver	8	1	-
Bone	7	3	-
**Histopathology (n; %)**			
Invasive ductal carcinoma (IDC)	84 (84.8%)	5 (71.4%)	0.353
Invasive lobular carcinoma (ILC)	5 (5.1%)	0 (0.0%)	0.542
Mixed IDC/ILC	7 (7.1%)	1 (14.3%)	0.489
Others (e.g., **mucinous**)	1 (1.0%)	0 (0.0%)	0.791
Unknown	2 (2.0%)	1 (14.3%)	0.058
**Tumor grade (n; %)**			
Grade I	10 (10.1%)	0 (0.0%)	0.379
Grade II	36 (36.4%)	2 (28.6%)	0.679
Grade III	39 (39.4%)	4 (57.1%)	0.359
Unknown	14 (14.1%)	1 (14.3%)	0.988
**Immunohistochemistry (n; %)**			
Luminal A	9 (9.1%)	1 (14.3%)	0.651
Luminal B HER2−	27 (27.3%)	1 (14.3%)	0.453
Luminal B HER2+	19 (19.2%)	1 (14.3%)	0.750
HER2 **enriched**	16 (16.2%)	0 (0.0%)	0.250
HER2 **low**	6 (6.1%)	1 (14.3%)	0.402
TNBC	7 (7.1%)	1 (14.3%)	0.489
Unknown	15 (15.2%)	2 (28.6%)	0.353
**Pharmacotherapy phase (n; %)**			
Neoadjuvant	35 (35.4%)	1 (14.3%)	0.257
Adjuvant	39 (39.4%)	1 (14.3%)	0.188
Palliative	25 (25.3%)	5 (71.4%)	0.009 *
**Pharmacotherapy (n)**			
Paclitaxel—Carboplatin	8	0	-
Docetaxel—Carboplatin	18	2	-
Docetaxel—Cyclophosphamide	4	0	-
Doxorubicin—Cyclophosphamide	17	0	-
Docetaxel—Doxorubicin—Cyclophosphamide	11	1	-
Docetaxel—Epirubicin—Cyclophosphamide	3	2	-
Epirubicin—Paclitaxel	1	0	-
Epirubicin—Docetaxel	1	0	-
Epirubicin—Carboplatin	3	0	-
Epirubicin—Cyclophosphamide—5FU	3	0	-
Cyclophosphamide—Methotrexate—5FU	1	0	-
Paclitaxel—Trastuzumab	2	0	-
Docetaxel—Trastuzumab	2	0	-
Paclitaxel—Carboplatin—Trastuzumab	2	0	-
Docetaxel—Carboplatin—Trastuzumab	11	0	-
Epirubicin—Docetaxel—Trastuzumab	1	0	-
Trastuzumab	1	1	-
Carboplatin—Gemcitabine	1	0	-
Paclitaxel—Gemcitabine	1	0	-
Docetaxel—Capecitabine	1	0	-
Vinorelbine	1	2	-
Eribulin	1	0	-
Leuprorelin	1	0	-
Tamoxifen	2	0	-
Fulvestrant	1	0	-
Letrozole	3	0	-
Goserelin	7	0	-
Bevacizumab	2	0	-
Zolendronic acid	5	0	-

^#^ Comparisons between proportions were performed using the Chi-Square Test of Independence. * statistically significant at *p* < 0.05. n = number of patients; % = percentage; IDC = invasive ductal carcinoma; ILC = invasive lobular carcinoma; HER2 = human epidermal growth factor receptor 2; TNBC = triple-negative breast cancer; FU = 5-fluorouracil.

**Table 3:** Comparison of pre-pharmacotherapy quality-of-life scores between survived and non-survived patients.

Parameter	Survived Median [IQR] (N = 99)	Non-Survived Median [IQR] (N = 7)	*p*-Value *^#^*
**Global health status**			
QoL (QL2)	83.3 [66.7–91.7]	41.7 [25.0–50.0]	<0.001 *
**C30—Functional scale**			
Physical function (PF2)	93.3 [80.0–100.0]	66.7 [6.7–100.0]	0.027 *
Role function (RF2)	100.0 [100.0–100.0]	66.7 [16.7–100.0]	0.009 *
Emotional function (EF)	83.3 [66.7–100.0]	41.7 [33.3–75.0]	0.002 *
Cognitive function (CF)	100.0 [83.3–100.0]	100.0 [100.0–100.0]	0.362
Social function (SF)	100.0 [100.0–100.0]	66.7 [33.3–83.3]	<0.001 *
**C30—Symptom scale**			
Fatigue (FA)	22.2 [0.0–44.4]	55.6 [0.0–77.8]	0.107
Nausea vomiting (NV)	0.0 [0.0–0.0]	0.0 [0.0–16.7]	0.627
Pain (PA)	16.7 [16.7–33.3]	66.7 [33.3–83.3]	0.008 *
Dyspnea (DY)	0.0 [0.0–0.0]	0.0 [0.0–66.7]	0.093
Insomnia (SL)	33.3 [0.0–66.7]	66.7 [0.0–100.0]	0.257
Appetite loss (AP)	0.0 [0.0–33.3]	33.3 [0.0–66.7]	0.010 *
Constipation (CO)	0.0 [0.0–0.0]	33.3 [0.0–33.3]	0.010 *
Diarrhea (DI)	0.0 [0.0–0.0]	0.0 [0.0–0.0]	0.219
Financial difficulty (FI)	0.0 [0.0–33.3]	33.3 [0.0–100.0]	0.047 *
**BR23—Functional scale**			
Body image (BRBI)	100.0 [83.3–100.0]	66.7 [41.7–100.0]	0.234
Sexual function (BRSEF)	33.3 [0.0–33.3]	33.3 [0.0–33.3]	0.720
Sexual enjoyment (BRSEE)	33.3 [0.0–33.3]	33.3 [0.0–33.3]	0.896
Future perspective (BRFU)	66.7 [33.3–100.0]	33.3 [0.0–33.3]	0.014 *
**BR23—Symptom scale**			
Systemic side effect (BRST)	9.5 [4.8–19.0]	23.8 [14.3–33.3]	0.037 *
Breast symptom (BRBS)	16.7 [8.3–25.0]	8.3 [0.0–41.7]	0.722
Arm symptom (BRAS)	11.1 [0.0–33.3]	44.4 [22.2–55.6]	0.050 *
Upset by hair loss (BRHL)	0.0 [0.0–0.0]	0.0 [0.0–0.0]	0.931

^#^ Comparisons between two independent (unpaired) groups were performed using the Mann–Whitney U test for independent samples. * statistically significant at *p* < 0.05. QL2 = global health status/quality of life; PF2 = physical functioning; RF2 = role functioning; EF = emotional functioning; CF = cognitive functioning; SF = social functioning; FA = fatigue; NV = nausea and vomiting; PA = pain; DY = dyspnea; SL = insomnia; AP = appetite loss; CO = constipation; DI = diarrhea; FI = financial difficulty; BRBI = body image; BRSEF = sexual functioning; BRSEE = sexual enjoyment; BRFU = future perspective; BRST = systemic therapy side effects; BRBS = breast symptoms; BRAS = arm symptoms; BRHL = upset by hair loss; IQR = interquartile range.

**Table 4:** Area under the curve of EORTC QLQ-C30 global health status and functional domains for prediction of first-cycle pharmacotherapy survival.

C30—Functional Scale	Area Under the Curve (AUC)	*p*-Value
QL2	0.907	0.000 *
PF2	0.737	0.037 *
RF2	0.718	0.055
EF	0.846	0.002 *
SF	0.843	0.003 *

* statistically significant at *p* < 0.05. QL2 = global health status/quality of life; PF2 = physical functioning; RF2 = role functioning; EF = emotional functioning; SF = social functioning; C30 = EORTC QLQ-C30 questionnaire; AUC = area under the curve; *p* = probability value.

**Table 5:** Area under the curve of EORTC QLQ-C30 symptom domains for prediction of first-cycle pharmacotherapy survival.

C30—Symptom Scale	Area Under the Curve (AUC)	*p*-Value
PA	0.208	0.010 *
AP	0.266	0.039 *
CO	0.304	0.085
FI	0.320	0.112

* statistically significant at *p* < 0.05. PA = pain; AP = appetite loss; CO = constipation; FI = financial difficulty; C30 = EORTC QLQ-C30 questionnaire; AUC = area under the curve; *p* = probability value.

**Table 6:** Area under the curve of EORTC QLQ-BR23 domains for prediction of first-cycle pharmacotherapy survival.

QLQ-BR23	Area Under the Curve (AUC)	*p*-Value
BRFU	0.768	0.018 *
BRST	0.266	0.039 *
BRAS	0.284	0.056

* statistically significant at *p* < 0.05. BRFU = future perspective; BRST = systemic therapy side effects; BRAS = arm symptoms; QLQ-BR23 = breast cancer–specific module of the EORTC quality of life questionnaire; AUC = area under the curve; *p* = probability value.

**Table 7:** Sensitivity and specificity of EORTC QLQ-C30 global health status (QL2) across different cut-off values for predicting first-cycle pharmacotherapy survival.

Test Result Variable	Cut-Off Value	Sensitivity	1 − Specificity	Youden Index
QL2	−1.0000	1.000	1.000	0.000
	12.5000	0.990	1.000	−0.010
	29.1500	0.980	0.714	0.266
	33.3167	0.970	0.714	0.256
	37.5000	0.960	0.571	0.389
	45.8333	0.949	0.429	0.520
	54.1667	0.879	0.143	0.736
	62.5000	0.818	0.143	0.675
	70.8333	0.707	0.143	0.564
	79.1667	0.556	0.000	0.556
	87.5000	0.293	0.000	0.293
	95.8333	0.172	0.000	0.172
	101.0000	0.000	0.000	0.000

## Data Availability

The data that support the findings of this study are available on request from the corresponding author H.S.

## Abbreviations

AP	Appetite Loss
AUC	Area Under the Curve
BR23	Breast Cancer–Specific Module of the EORTC QLQ
BRAS	Arm Symptoms
BRBI	Body Image
BRBS	Breast Symptoms
BRFU	Future Perspective
BRHL	Upset by Hair Loss
BRSEE	Sexual Enjoyment
BRSEF	Sexual Functioning
BRST	Systemic Therapy Side Effects
C30	Core 30 Questionnaire of the EORTC QLQ
CF	Cognitive Functioning
CO	Constipation
DI	Diarrhea
DY	Dyspnea
EF	Emotional Functioning
EORTC	European Organisation for Research and Treatment of Cancer
ER	Estrogen Receptor
FA	Fatigue
FI	Financial Difficulty
HER2	Human Epidermal Growth Factor Receptor 2
IDC	Invasive Ductal Carcinoma
IHC	Immunohistochemistry
ILC	Invasive Lobular Carcinoma
IQR	Interquartile Range
LMICs	Low- and Middle-Income Countries
NV	Nausea and Vomiting
PA	Pain
PF2	Physical Functioning
PR	Progesterone Receptor
PROs	Patient-Reported Outcomes
QL2	Global Health Status/Quality of Life
QLQ	Quality of Life Questionnaire
RF2	Role Functioning
ROC	Receiver Operating Characteristic
SF	Social Functioning
SL	Insomnia
TNBC	Triple-Negative Breast Cancer
